# Structural Transitions of the Conserved and Metastable Hantaviral Glycoprotein Envelope

**DOI:** 10.1128/JVI.00378-17

**Published:** 2017-10-13

**Authors:** Ilona Rissanen, Robert Stass, Antra Zeltina, Sai Li, Jussi Hepojoki, Karl Harlos, Robert J. C. Gilbert, Juha T. Huiskonen, Thomas A. Bowden

**Affiliations:** a Division of Structural Biology, Wellcome Trust Centre for Human Genetics, University of Oxford, Oxford, United Kingdom; b Institute of Veterinary Pathology, Vetsuisse Faculty, University of Zürich, Zürich, Switzerland; c Department of Virology, Medicum, University of Helsinki, Helsinki, Finland; d Department of Biosciences, University of Helsinki, Helsinki, Finland; University of Utah

**Keywords:** X-ray crystallography, bunyavirus, cryo-EM, hantavirus, host cell infection, structural biology, viral glycoprotein, virus structure

## Abstract

Hantaviruses are zoonotic pathogens that cause severe hemorrhagic fever and pulmonary syndrome. The outer membrane of the hantavirus envelope displays a lattice of two glycoproteins, Gn and Gc, which orchestrate host cell recognition and entry. Here, we describe the crystal structure of the Gn glycoprotein ectodomain from the Asiatic Hantaan virus (HTNV), the most prevalent pathogenic hantavirus. Structural overlay analysis reveals that the HTNV Gn fold is highly similar to the Gn of Puumala virus (PUUV), a genetically and geographically distinct and less pathogenic hantavirus found predominantly in northeastern Europe, confirming that the hantaviral Gn fold is architecturally conserved across hantavirus clades. Interestingly, HTNV Gn crystallized at acidic pH, in a compact tetrameric configuration distinct from the organization at neutral pH. Analysis of the Gn, both in solution and in the context of the virion, confirms the pH-sensitive oligomeric nature of the glycoprotein, indicating that the hantaviral Gn undergoes structural transitions during host cell entry. These data allow us to present a structural model for how acidification during endocytic uptake of the virus triggers the dissociation of the metastable Gn-Gc lattice to enable insertion of the Gc-resident hydrophobic fusion loops into the host cell membrane. Together, these data reveal the dynamic plasticity of the structurally conserved hantaviral surface.

**IMPORTANCE** Although outbreaks of Korean hemorrhagic fever were first recognized during the Korean War (1950 to 1953), it was not until 1978 that they were found to be caused by Hantaan virus (HTNV), the most prevalent pathogenic hantavirus. Here, we describe the crystal structure of HTNV envelope glycoprotein Gn, an integral component of the Gn-Gc glycoprotein spike complex responsible for host cell entry. HTNV Gn is structurally conserved with the Gn of a genetically and geographically distal hantavirus, Puumala virus, indicating that the observed α/β fold is well preserved across the Hantaviridae family. The combination of our crystal structure with solution state analysis of recombinant protein and electron cryo-microscopy of acidified hantavirus allows us to propose a model for endosome-induced reorganization of the hantaviral glycoprotein lattice. This provides a molecular-level rationale for the exposure of the hydrophobic fusion loops on the Gc, a process required for fusion of viral and cellular membranes.

## INTRODUCTION

Viruses in the family Hantaviridae, within the order Bunyavirales, comprise a group of negative-sense, single-stranded RNA viruses (1, 2). Hantaviruses known to be pathogenic in humans belong to three clades, each carried by a distinct group of rodents: Old World mice and rats (Murinae), New World mice and rats (Neotominae and Sigmodontinae), and voles (Arvicolinae). Upon zoonotic transmission into humans, hantaviruses cause hemorrhagic fever with renal syndrome (HFRS) or hantavirus cardiopulmonary syndrome (HCPS), diseases resulting in mortality rates of up to 12% and 40%, respectively (1, 3, 4). While humans are typically infected via inhalation of aerosolized excreta from rodent carriers, human-to-human transmission has been reported for the South American Andes virus (ANDV) (5, 6). Hantaviral infections affect tens of thousands of people annually, yet treatment and prevention options remain extremely limited (1).

The hantaviral envelope comprises a lipid bilayer with an outer proteinaceous shell of two glycoproteins, Gn and Gc, which are synthesized as a single polyprotein precursor (GPC) (7). Following cleavage of the GPC at a WAASA recognition sequence during protein biosynthesis (8), the Gn and Gc form a lattice of mature spike complexes (9–12). Crystal structures of Puumala virus (PUUV) Gn and Gc and Hantaan virus (HTNV) Gc ectodomains have been determined, revealing that the Gn forms a globular α/β sandwich and that the Gc forms a class II fusion protein fold, which forms trimers in the postfusion state (12–14). Fitting of the PUUV Gn ectodomain crystal structure into a low-resolution reconstruction of the mature hantaviral glycoprotein spike complex revealed that the Gn is membrane distal and likely shields hydrophobic fusion loops on the Gc (12). In addition to native functionality, hantaviral Gn and Gc glycoproteins are important targets of the neutralizing humoral immune response (15–19).

Several cellular factors, including integrins, decay-accelerating factor, and complement receptor gC1qR-p32, have been reported to mediate hantavirus entry into host cells (20–23). Following receptor recognition, hantaviruses undergo clathrin-mediated endocytosis, and the viral genome is delivered into the cytoplasm following Gc-mediated fusion of viral and host membranes, a process triggered by the acidic pH of late endosomes or lysosomes (24). Although histidines in Gc domain III have been shown to be essential for triggering Gc-mediated fusion in other bunyaviruses (25, 26), the residues involved in this pH-dependent process for hantaviruses have yet to be identified.

HTNV remains one of the most deadly and prevalent hantaviruses to infect humans, with approximately 1.5 million cases in China over the last six decades (1, 27, 28). Here, we sought to understand the architecture of the HTNV glycoprotein envelope and report the crystal structure of the Gn ectodomain to 2.15-Å resolution. HTNV Gn bears a striking resemblance to the genetically distal PUUV Gn, revealing that hantaviral glycoprotein structure and assembly are likely to be well-conserved across the Hantaviridae family (12). Furthermore, our HTNV Gn crystal structure was obtained at acidic pH and forms a tetrameric configuration architecturally distinct from its expected organization at neutral pH. Using analytical ultracentrifugation (AUC) and cryo-electron microscopy (cryo-EM), we confirmed that our Gn glycoprotein forms pH-sensitive oligomeric states in solution and that conformational rearrangements to the Gn-Gc glycoprotein lattice are incurred upon exposure to acidic environments, such as those existing in endosomal compartments. We present a model for the endosome-induced reorganization of the hantaviral glycoprotein lattice using the crystal structures of our tetrameric HTNV Gn and a previously reported HTNV Gc trimer (14) and propose that reorientation of the Gn provides the Gc with the steric freedom required for membrane fusion.

## RESULTS

### Structure of HTNV Gn.

**FIG 1:**
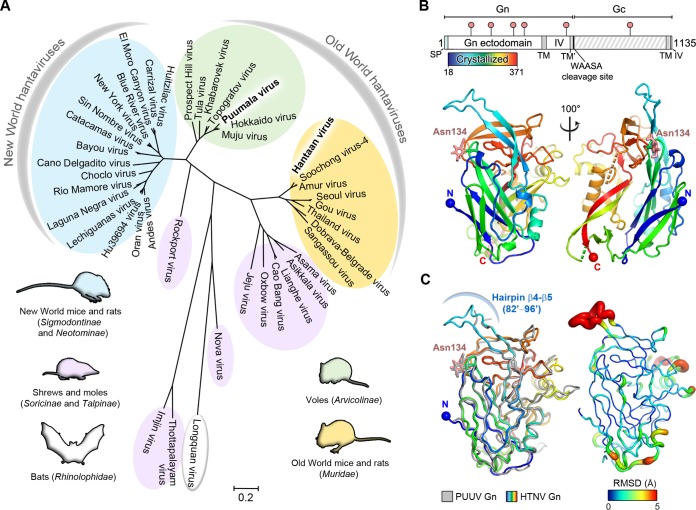
Phylogeny and crystal structure of HTNV Gn ectodomain. (A) A maximum-likelihood phylogeny of 42 hantaviral Gn glycoprotein sequences separates hantavirus species according to host reservoir. The three clades of hantaviruses borne by rodents are annotated in yellow, green, and blue. Hantaviruses carried by shrews and moles are annotated in purple, and Longquan virus, isolated from bats, is annotated in white. The scale bar indicates amino acid substitutions per site. (B) The organization of the HTNV glycoprotein precursor (above) and crystal structure of the HTNV Gn ectodomain to 2.15-Å resolution (below). The schematic was produced with DOG (60) with the crystallized region of the Gn indicated by a bar colored as a rainbow. The signal peptide (SP), transmembrane domains (TM), the hydrophobic region preceding the WAASA cleavage site (TM′), intraviral domains (IV), and WAASA signal peptidase cleavage site are annotated. Putative N-linked glycosylation sequons are labeled above the schematic (pink pins). The structure is presented as a cartoon and colored as a rainbow ramped from blue (N terminus) to red (C terminus). Disordered regions comprising residues 190 to 197 and residues 281 to 289 are highlighted (dotted lines, green and orange, respectively). The crystallographically observed glycan at Asn134 is shown as pink sticks. (C) Structural comparison of HTNV Gn and PUUV Gn. Overlay of HTNV Gn and PUUV Gn, colored as a rainbow and in gray, respectively (ribbon representation), is shown on the left. At right is the HTNV Gn with root mean square (RMS) deviation of equivalent residues between PUUV Gn mapped onto the Cα trace. The tube radius and color represent the RMS deviation (ramped from blue to red). Regions with high deviations between PUUV Gn and HTNV Gn structures are thick and red. Regions with low deviations are thin and blue.

Pathogens known to cause human disease within the family Hantaviridae are categorized into three clades according to their respective rodent reservoirs (voles, Old World mice and rats, and New World mice and rats) (Fig. 1A). Of the two glycoproteins presented on the hantaviral surface, the Gn is more exposed than the cognate Gc glycoprotein and exhibits higher levels of sequence diversity (12, 29). To determine whether the genetic diversity of the Gn glycoprotein is reflected in architecture, we sought to determine the structure of the Gn from HTNV, a pathogen responsible for severe HFRS in eastern Asia, and compared it to the previously reported structure of the Gn glycoprotein from the genetically distinct PUUV.

A construct comprising HTNV Gn ectodomain residues 18 to 371 (Fig. 1B) was crystallized under a condition comprising equal volumes of the protein in sample buffer (10 mM Tris, pH 8.0, 150 mM NaCl) and precipitant (1.6 M ammonium sulfate, 0.1 M citrate pH 4.0). The structure of HTNV Gn was solved to 2.15-Å resolution (Table 1) using the only other known hantaviral Gn crystal structure, PUUV Gn (PDB accession code 5FXU), as a search model. The one molecule of HTNV Gn observed in the asymmetric unit displays a β-sandwich fold stabilized by seven disulfide bonds and composed of five antiparallel β-sheets and six α-helices (Fig. 1B). While PUUV Gn and HTNV Gn share limited amino acid sequence identity (44%) and similarity (64%) over the crystallized region, HTNV Gn exhibits a high level of structural homology to PUUV Gn (1.3-Å root-mean-square deviation [RMSD] over 315 Cα atoms), corroborating the hypothesis that the Gn fold is a well-conserved feature among hantaviruses (Fig. 1C) (12). We note that two loops (residues 190 to 197 and residues 281 to 289) (Fig. 1B) were not ordered well enough for model building in the HTNV Gn structure. These loops are also disordered in PUUV Gn, indicating inherent flexibility or a requirement for an additional interaction partner (e.g., the cognate Gc) to assume a stable conformation.

**TABLE 1:** Crystallographic data and refinement statistics for HTNV Gn

Parameter	Value for the parameter^*a*^
Data collection statistics	
Space group	*I*422
Cell dimensions	
*a*, *b*, *c* (Å)	110.9, 110.9, 180.6
α, β, γ (°)	90.0, 90.0, 90.0
Resolution range (Å)	78.42–2.15 (2.21–2.15)
*R*_merge_	0.195 (>1)
*I*/σ(*I*)	14.8 (2.0)
CC_1/2_^*b*^	0.999 (0.883)
Completeness (%)	100 (100)
Redundancy	38.2 (36.4)
Refinement statistics	
Resolution (Å)	59.21–2.15 (2.22–2.15)
No. reflections	30,848 (1,574)
*R*_work_/*R*_free_	0.201/0.227
No. of atoms	
Protein	2,588
Ligand/ion	48
Water	171
B factors (Å^2^)	
Protein	48.1
Ligand/ion	68.9
Water	47.1
RMS deviation	
Bond length (Å)	0.002
Bond angle (°)	0.447
Ramachandran statistics (%)	
Residues in preferred region	96.04
Residues in allowed region	3.96
Outliers	0

a The value for the highest-resolution shell is shown in parentheses.

b The Pearson correlation coefficient is calculated between two random half data sets.

**FIG 2:**
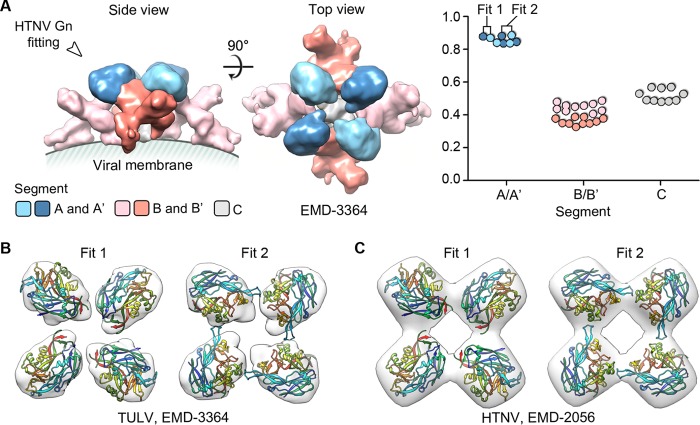
Fitting of HTNV Gn into the TULV glycoprotein spike localizes the Gn to tetrameric membrane distal lobes. (A) The TULV glycoprotein spike (Electron Microscopy Data Bank [EMDB] accession no. EMD-3364) was partitioned into five unique segments, as previously described (12): two globular membrane-distal volumes (segment A and A′, light and dark blue, respectively), two elongated volumes (segment B and B′, pink and salmon, respectively), and a central stalk (segment C, gray), using Segger (58). Fitting analysis, using the fit-to-segments function of Segger, reveals that HTNV Gn most likely localizes to the membrane-distal volumes, as shown in the plot (right) that illustrates the goodness of fit (density occupancy score) of HTNV Gn into the density segments. The two highest-scoring fitting outcomes for Gn are shown within the globular membrane-distal volumes of TULV (12) (B) and HTNV (10) (C) cryo-EM reconstructions.

Consistent with a previous fitting of PUUV Gn into the highest resolution cryo-electron microscopy reconstruction of a hantaviral envelope available (Tula virus [TULV], 16-Å resolution) (12), fitting of individual HTNV Gn protomers reveals that HTNV Gn likely localizes to membrane-distal tetrameric spikes of the mature envelope surface (Fig. 2). We observed two potential orientations (Fig. 2, fit 1 and fit 2) of the HTNV Gn in this membrane-distal density, both of which were also congruent with a lower-resolution (25-Å) reconstruction of the HTNV glycoprotein spike (10). This compatibility of fit suggests that despite the observed differences in the TULV and HTNV reconstructions (7, 10, 12), which may potentially have resulted from the different averaging techniques employed, genetically distinct hantaviral Gn glycoprotein ectodomains are likely to assume a conserved tetrameric and membrane-distal configuration, shielding the Gc fusion protein on the mature virion.

### A potential HTNV Gn homo-oligomerization interface formed at acidic pH.

**FIG 3:**
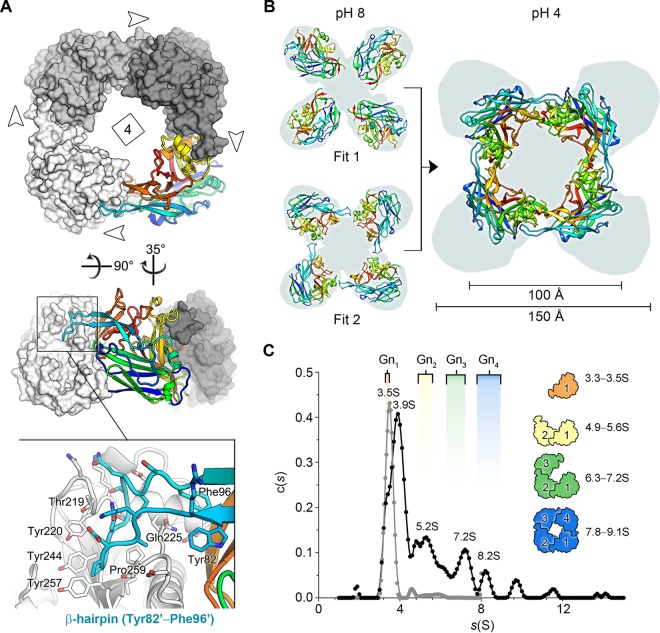
The acidic tetramer of HTNV Gn presents an oligomeric conformation distinct from that predicted at neutral pH. (A) Structure of the crystallographically observed HTNV Gn tetramer. Three protomers are colored in shades of gray in a surface representation. The fourth Gn protomer is shown as a cartoon and colored as described in the legend to Fig. 1B. Insets show that the interaction interface is facilitated by the Tyr82-Phe96 β-hairpin loop, which interlocks into a pocket formed in the adjacent protomer. (B) Comparison of the tetramers formed from the two highest scoring fits, presented in Fig. 2, with that of the crystallographically observed acidic tetramer. On the left-hand side, the two highest scoring fits are shown (cartoon representation) with the membrane-distal volumes of the TULV spike reconstruction (EMDB accession no. EMD-3364) shown as a gray background. The right-hand side shows the acidic tetramer placed in the same position, revealing that that the span of the assembly is approximately 50 Å less than the width of the tetramer observed at physiological pH. (C) Analytical ultracentrifugation analysis reveals that HTNV Gn forms higher-order oligomeric states in solution upon acidification, including putative dimers, trimers, and tetramers. A sedimentation coefficient distribution plot shows populations of HTNV Gn at pH 7.0 (in gray) and 4.5 (in black). Experimentally derived sedimentation coefficient values (Svedberg units [S]) are shown above each peak. For comparison, theoretical S values, which were calculated using the crystallographically observed acidic tetramer and derivative monomer, dimer, and trimer subcomponents, are shown with equivalent subunit representations to the right. The range of values represents variable possible glycosylation states of the Gn protein: the first value is calculated without glycans modeled, and the second value is calculated with high-mannose-type glycans (61) modeled at N-linked glycosylation sites as rigid side chains. This analysis revealed that the experimental peaks at 3.5 to 3.9S, 5.2S, 7.2S, and 8.2S closely approximate the expected migration of a monomer, dimer, trimer, and tetramer, respectively. In addition, minor populations of larger HTNV Gn oligomers were detected. Theoretical S values were calculated using SoMo (62–64) with the optimal overlap bead model approach and hydrodynamic parameter determination with ZENO (62).

HTNV Gn was crystallized at acidic pH, and analysis of crystallographic packing revealed a compact tetrameric assembly, termed herein the acidic Gn tetramer (Fig. 3). Residues 82 to 96 appear to play a chief role in the construction of this higher-order assembly, forming a β-hairpin that interlocks into a pocket formed on the neighboring Gn protomer (Fig. 3A). Each of the four equivalent protomeric interfaces of the acidic Gn tetramer is extensive, occluding approximately 1,850 Å^2^ of solvent-accessible surface, and reinforced by 16 hydrogen bonds (30).

The formation of a homotetrameric Gn in the crystal is consistent with the existence of tetrameric Gn-Gc spikes, as displayed on the hantaviral surface (9, 12). However, we note that our acidic tetramer is architecturally distinct from that formed by fitting of individual Gn glycoproteins into a reconstruction of the mature (pH 8.0) TULV spike (Fig. 2B and 3B). Indeed, although the acidic tetramer more closely resembles fit 2 than fit 1, it is far more compact than either fit and exhibits a width reduced by approximately one-third (50 Å) (Fig. 3B). The conformation of the acidic tetramer thus differs from that existing on the mature virion, raising the possibility that it may be relevant during endocytosis of the virion into a host cell.

### HTNV Gn forms higher-order oligomeric assemblies at acidic pH.

We have previously shown by size exclusion chromatography (SEC) analysis that recombinantly produced hantaviral Gn proteins are monomeric in solution at low concentrations at both neutral and acidic pH (12). To assess the oligomeric state of soluble Gn at higher concentrations, analogous to those observed on the native viral envelope (12), we studied HTNV Gn by AUC under both pH-neutral (7.0) and acidic (4.5) conditions. Consistent with our previous SEC analysis, HTNV Gn was predominantly monomeric in solution at pH 7.0 (sedimentation coefficient *s* in Svedberg units [S] = 3.5) (Fig. 3C). Acidification of the Gn, by contrast, resulted in the formation of an array of higher-order species, including putative dimers (S = 5.2), trimers (S = 7.2), and tetramers (S = 8.2). The formation of these species suggests that higher-order Gn oligomers, such as the acidic tetramer observed in our HTNV Gn crystal structure, may form on the hantaviral surface during endocytosis.

### Gn-Gc lattices are disrupted upon exposure to acidic pH.

**FIG 4:**
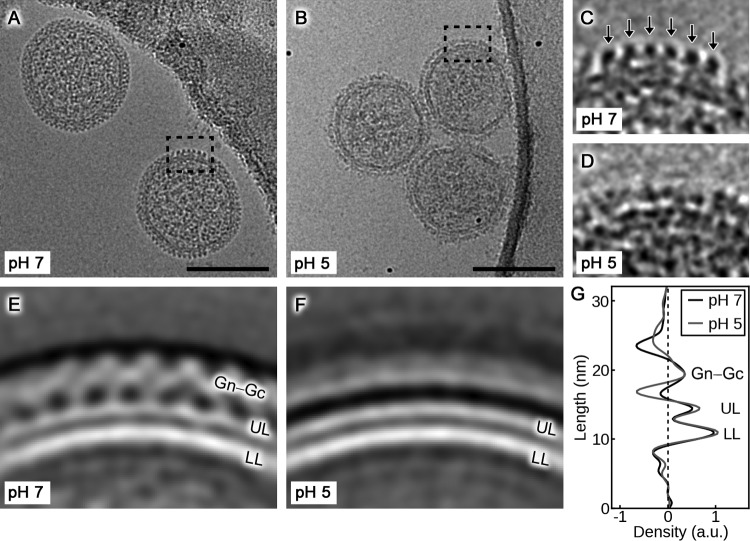
Cryo-electron microscopy analysis reveals the metastability of the hantavirus envelope. (A and B) Representative cryo-electron micrographs of TULV after incubation at pH 7 and pH 5, respectively. Scale bar, 100 nm. (C and D) Close-ups of the areas indicated in panels A and B with the regular lattice of Gn-Gc spikes indicated with arrows in panel C. No regular lattice is visible in the virions incubated at pH 5. (E and F) Representative 2D class averages of the virion surface at pH 7 and pH 5. Density has been inverted so that white corresponds to the virion. (G) An averaged density profile of the images in panels E and F indicating the average distribution of density perpendicular to the membrane at pH 7 and pH 5. Densities corresponding to the lower membrane leaflet (LL), the upper membrane leaflet (UL), and Gn-Gc spikes are indicated. a.u., arbitrary units.

Although the pH-dependent conformational rearrangements of the hantaviral Gc fusion glycoprotein are well established (13, 14, 31), there exists a paucity of information with respect to the changes that occur to the higher-order Gn-Gc spike complex upon endocytic uptake of the virion. Using nonpathogenic TULV as a model, we utilized cryo-EM to establish whether proton-induced changes to hantaviral glycoprotein ultrastructure are compatible with our crystallographically observed acidic Gn tetramer (Fig. 4).

As expected (12), analysis of TULV particles at neutral pH revealed a regular lattice of two distinct Gn-Gc layers encapsulating a roughly spherical lipid bilayer envelope (Fig. 4A, C, and E). Analysis of TULV envelope at acidic pH, on the other hand, demonstrated that although the virion lipid bilayer maintains a level of regularity similar to that at neutral pH, the Gn-Gc spike complex is metastable and undergoes dramatic structural changes, including the dissolution of the Gn-Gc lattice (Fig. 4B, D, F, and G). These data indicate that even in the absence of native host cell factors, such as endosomal components (32, 33), virion acidification is sufficient to trigger hantaviral glycoprotein reorganization, most likely reconfiguring homotypic and heterotypic interactions between Gn and Gc in the process.

## DISCUSSION

Here, we present the crystal structure of the Gn glycoprotein from the highly pathogenic HTNV from the family Hantaviridae within the order Bunyavirales (Fig. 1B). Docking of our HTNV Gn crystal structure into a previously reported hantavirus cryo-EM reconstruction localizes the Gn to the outermost surface of the viral envelope (Fig. 2). Similar to studies of Bunyamwera virus in the related Peribunyaviridae family, genus Orthobunyavirus (34), treatment of recombinantly derived Gn glycoprotein and live hantavirions with acidic buffer revealed that the configuration of the envelope glycoprotein complex is metastable and likely undergoes dramatic conformational transitions upon exposure to endosomal compartments (Fig. 3 and 4).

**FIG 5:**
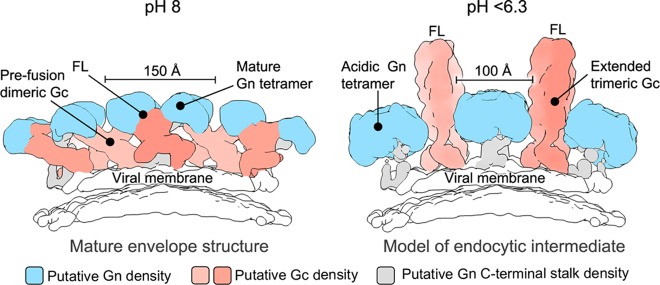
A model of pH-dependent conformational changes to the Gn and Gc glycoproteins during endocytosis, prior to fusion. At left is a side-view schematic of the mature hantavirus envelope, based upon a previously reported reconstruction of the TULV glycoprotein spike (EMDB accession no. EMD-3364). The globular Gn glycoprotein (blue), Gc glycoprotein (salmon), C terminus of the Gn-glycoprotein (gray), and putative location of the Gc fusion loops (FL; buried in the Gn-Gc interface), are indicated. Disassembly of the Gn-Gc spike complex (right panel) and the formation of the acidic Gn tetramer could create the space necessary for trimerization of the Gc glycoprotein with the fusion loops facing outwards to enable contact with target cellular membranes. The Gc trimer shown in the schematic is based on the HTNV Gc crystal structure (PDB accession number 5LJZ) (14), with domain III rotated 90° to model an extended class II fusion protein trimer that has been suggested to exist at low pH, prior to membrane fusion (35, 39). The width of the putative Gn tetramers formed under both pH-neutral and acidic conditions is annotated.

Disruption of heterotypic glycoprotein contacts is required for Gc homotrimerization and insertion of Gc-resident hydrophobic fusion loops into the host cell membrane (13, 14, 31, 35). We present a model predicting how the Gn both shields the hydrophobic fusion loops on the Gc at neutral pH and dissociates from the Gc during Gc-mediated fusion in endosomal compartments (Fig. 5). This model is also consistent with the formation of our crystallographically observed acidic Gn tetramer (Fig. 3), which would sterically facilitate this process by both (i) detaching from the Gc, thus exposing the hydrophobic fusion loops, and (ii) forming a narrower configuration that would provide the freedom for Gc to undergo dimer-to-trimer transitions, including the formation of an expected extended intermediate.

On a broader level, although bunyaviral glycoprotein spike complexes are similarly derived from two components, Gn and Gc, cryo-EM studies have revealed that the glycoproteins form strikingly diverse assemblies (34). Indeed, the higher-order architecture of the Gn-Gc spike ranges from orthobunyaviral Gn-Gc tripods (34) to hantaviral Gn-Gc tetramers (10–12) and phleboviral icosahedrons composed of Gn-Gc pentamers and hexamers (36–38). Nonetheless, crystallographic investigations of the Gc glycoprotein have revealed commonalities, where hantaviral and phleboviral Gc glycoproteins (13, 14, 25, 39) exhibit a functionally and structurally conserved class II fusion protein fold, which is also present in genetically and patho-physiologically distinct alphaviruses, flaviviruses, and even eukaryotes (40, 41).

The level of structural conservation of the bunyaviral Gn glycoprotein, on the other hand, is more enigmatic. Although the mixed α/β fold of the hantaviral Gn ectodomain resembles the alphaviral E2 glycoprotein (42), supporting a conserved functional role in guarding the hydrophobic fusion loops displayed by the cognate fusion glycoprotein, the hantaviral Gn and alphaviral E2 differ substantially. Structural analyses of Gn glycoproteins from other bunyaviral families will therefore be enlightening as to the origin(s) of the observed mixed α/β Gn fold. Furthermore, if the Gn glycoprotein is the more structurally variable component of the bunyaviral Gn-Gc spike complex, it may be the architectural mortar which assembles with the conserved Gc building blocks into the diverse glycoprotein spike assemblies reported across the Bunyavirales.

## MATERIALS AND METHODS

### Molecular phylogenetic analysis by the maximum-likelihood method.

The evolutionary history was inferred by using the maximum-likelihood method based on the model of Le and Gascuel (43). The tree with the highest log likelihood is shown (Fig. 1A). Initial tree(s) for the heuristic search were obtained automatically by applying neighbor-joining and BioNJ algorithms to a matrix of pairwise distances estimated using a Jones-Taylor-Thornton (JTT) model and then selecting the topology with the superior log-likelihood value. A discrete gamma distribution (+G) was used to model evolutionary rate differences among sites (5 categories [+G; parameter = 1.2653]). The rate variation model allowed for some sites to be evolutionarily invariable ([+I], 7.9248% sites). The tree is drawn to scale, with branch lengths measured in the number of substitutions per site. The analysis involved 42 hantavirus Gn amino acid sequences. All positions containing gaps and missing data were eliminated. There were a total of 612 positions in the final data set. Evolutionary analyses were conducted in MEGA7 (44).

### Expression and purification of HTNV Gn.

The N-terminal ectodomain of HTNV Gn (residues 18 to 371; GenBank accession number AIL25321.1) was PCR-amplified from codon-optimized cDNA (GeneArt, Life Technologies) and cloned into the pHLsec mammalian expression vector (45). The resulting plasmid cDNA was transiently transfected in human embryonic kidney (HEK) 293T cells (ATCC CRL-1573), as previously described (45), in the presence of the class 1 α-mannosidase inhibitor, kifunensine (46). Cell supernatants were harvested and subjected to diafiltration at 90 h posttransfection (AKTA Flux diafiltration system; GE Healthcare), and HTNV Gn was purified by immobilized metal affinity chromatography (5-ml fast flow crude column and ÄKTA fast protein liquid chromatography [FPLC] system; GE Healthcare) followed by size exclusion chromatography using a Superdex 200 10/300 Increase column (GE Healthcare), in 10 mM Tris (pH 8.0)–150 mM NaCl buffer.

### Crystal structure determination.

Prior to crystallization, purified HTNV Gn was deglycosylated with endoglycosidase F1 (endo F1; 0.01 mg of endo F1 per 1 mg of HTNV Gn; 18 h of incubation at 21°C) and repurified by size exclusion chromatography using a Superdex 200 Increase 10/300 GL column (GE Healthcare) in 10 mM Tris (pH 8.0)–150 mM NaCl buffer. Purified HTNV Gn was crystallized at room temperature using the sitting-drop vapor diffusion method (47) using 100 nl of protein (6.3 mg/ml, in 10 mM Tris, pH 8.0, 150 mM NaCl buffer) plus 100 nl of precipitant containing 1.6 M ammonium sulfate and 0.1 M citrate at pH 4.0 after 132 days. The crystal was flash-frozen by immersion into a cryo-protectant containing the precipitant mixed with 25% (vol/vol) glycerol, followed by rapid transfer into liquid nitrogen. X-ray diffraction data were recorded on a Dectris Pilatus 6M-F detector at beamline i04 (wavelength [λ], 0.9795 Å), Diamond Light Source, United Kingdom.

Data were indexed, integrated, and scaled with XIA2 (48). The structure of HTNV Gn was solved by molecular replacement with the program Phaser-MR, within the PHENIX suite (49), using PUUV Gn (PDB accession number 5FXU [12]) as a search model. Structure refinement was performed using iterative refinement in PHENIX. Coot was used for manual rebuilding, and MolProbity was used to validate the model (50, 51). Processing statistics are presented in Table 1.

### AUC analysis.

The HTNV Gn ectodomain was purified as described above. Following purification, aliquots of the sample were buffer exchanged into (i) 10 mM Tris (pH 7.0)–150 mM NaCl and (ii) 10 mM citrate (pH 4.5)–150 mM NaCl. Protein samples were concentrated to 4.9 mg/ml, and sedimentation velocity AUC experiments were performed using a Beckman Optima XL-I operating at 20°C and a speed of 40,000 rpm. Sedimentation profiles were recorded using absorbance optics at a wavelength of 260 nm and interference optics every 6 min for a total of 120 scans. The data were analyzed using Sedfit software with *c*(*s*) (sedimentation coefficient distribution) and *c*(*s*, *f*/*f*_0_) (size-and-shape distribution) protocols (52).

### Virus preparation.

TULV (strain Moravia) was cultivated on Vero E6 cells (ATCC 94 C-RL1586), as previously described (11). Four days postinfection (dpi), the growth medium was replaced by medium supplemented with 3% fetal calf serum (FCS). At 5, 6, and 7 dpi the virus-containing medium was collected and clarified by centrifugation (3,270 × *g* for 30 min) to remove cell debris. The medium was then concentrated 100-fold using a 100-kDa-cutoff filter (Amicon) and placed on top of a 25 to 65% sucrose density gradient in standard buffer (20 mM Tris, pH 7.0, and 100 mM NaCl, pH 7.0) in an SW32.1 tube (Beckman Coulter), and the virus was banded by ultracentrifugation (24,000 rpm at 4°C for 12 h; SW32 Ti rotor). Virus-containing fractions were pooled and diluted 1:1 in standard buffer before being pelleted by ultracentrifugation (50,000 rpm at 4°C for 2 h; Beckman Coulter TLS 55 rotor). The virus pellet was resuspended in 40 μl of standard buffer.

### Electron microscopy.

Three microliters of TULV was applied to 1.2-μm-hole carbon grids (C-flat; Protochips) that were then floated on a droplet of either pH 7.0 or pH 5.0 buffer for 1 min prior to plunge freezing. The pH 7.0 buffer used was the standard buffer, and the pH 5.0 buffer was succinic acid, sodium dihydrogen phosphate, and glycine (SPG) in the molar ratio of 2:7:7, adjusted to pH 5.0 with HCl. Three microliters of 6-nm gold fiducial markers were added for tomography analysis. Image acquisition and processing data were collected using a Tecnai F30 Polar' transmission electron microscope (FEI) operated at 300 kV and at liquid nitrogen temperature. Serial EM (53) was used to acquire images on a direct electron detector (K2 Summit; Gatan) mounted behind an energy filter (QIF Quantum LS; Gatan) and operated at zero-energy-loss mode (slit width, 20 eV). Movies consisting of eight (pH 5.0) or 25 (pH 7.0) frames were acquired at a calibrated magnification of ×37,037, corresponding to a pixel size of 1.35 Å with a defocus target of 4 μm.

Movie frames were aligned and averaged to account for beam-induced motion and damage using MotionCor2 (54) prior to further processing with Relion (55). Contrast transfer function parameters were estimated using CTFFIND4 (56), and images were corrected by phase flipping. Particles were picked in an evenly spaced circle around the edge of each virus. The images were then binned by a factor of two and subjected to two-dimensional (2D) classification with a restricted angular search around the original in-plane angle calculated from the nearly circular geometry of the particle projections. Totals of 226 (pH 7.0) and 278 (pH 5.0) particles contributed to each final 2D average. A density profile along the direction perpendicular to the membrane was calculated in Bsoft (57).

### Fitting of the HTNV Gn structure into the TULV reconstruction.

The crystal structure of HTNV Gn was fitted in the TULV cryo-electron tomography (ET) reconstruction, as previously described for PUUV Gn (12). In short, the fitting was performed in the segmented density map of TULV using the fit-to-segments function of Segger (58) in Chimera (59). The crystal structure was converted to density by low-pass filtering to 16-Å resolution with 2.7-Å grid spacing. Subsequently, 1,000 evenly rotated fits were considered for each segment of the EM density while the density outside the target segment was masked. Similar to the fitting of PUUV Gn into the TULV map, fitting of HTNV Gn resulted in the highest density occupancy and cross-correlation values at the membrane-distal lobes of the spike. From the pool of 1,000 evenly rotated fits, the best scoring unique fits were generated by using the optimize fits option of Segger. Redundant fits less than 5 Å or 3° apart were discarded.

### Accession number(s).

Atomic coordinates and structure factors of HTNV Gn have been deposited in the Protein Data Bank (PDB) under accession number 5OPG.

## References

[1] Jonsson CB, Figueiredo LTM, Vapalahti O 2010 A global perspective on hantavirus ecology, epidemiology, and disease. Clin Microbiol Rev 23:412–441. doi:10.1128/CMR.00062-09. 20375360 PMC2863364

[2] Vaheri A, Strandin T, Hepojoki J, Sironen T, Henttonen H, Makela S, Mustonen J 2013 Uncovering the mysteries of hantavirus infections. Nat Rev Microbiol 11:539–550. doi:10.1038/nrmicro3066. 24020072

[3] Vaheri A, Henttonen H, Voutilainen L, Mustonen J, Sironen T, Vapalahti O 2013 Hantavirus infections in Europe and their impact on public health. Rev Med Virol 23:35–49. doi:10.1002/rmv.1722. 22761056

[4] Macneil A, Nichol ST, Spiropoulou CF 2011 Hantavirus pulmonary syndrome. Virus Res 162:138–147. doi:10.1016/j.virusres.2011.09.017. 21945215

[5] Martinez VP, Bellomo C, San Juan J, Pinna D, Forlenza R, Elder M, Padula PJ 2005 Person-to-person transmission of Andes virus. Emerg Infect Dis 11:1848–1853. doi:10.3201/eid1112.050501. 16485469 PMC3367635

[6] Martinez-Valdebenito C, Calvo M, Vial C, Mansilla R, Marco C, Palma RE, Vial PA, Valdivieso F, Mertz G, Ferres M 2014 Person-to-person household and nosocomial transmission of Andes Hantavirus, Southern Chile, 2011. Emerg Infect Dis 20:1629–1636. doi:10.3201/eid2010.140411. 25272189 PMC4193174

[7] Cifuentes-Munoz N, Salazar-Quiroz N, Tischler ND 2014 Hantavirus Gn and Gc envelope glycoproteins: key structural units for virus cell entry and virus assembly. Viruses 6:1801–1822. doi:10.3390/v6041801. 24755564 PMC4014721

[8] Lober C, Anheier B, Lindow S, Klenk HD, Feldmann H 2001 The Hantaan virus glycoprotein precursor is cleaved at the conserved pentapeptide WAASA. Virology 289:224–229. doi:10.1006/viro.2001.1171. 11689045

[9] Hepojoki J, Strandin T, Vaheri A, Lankinen H 2010 Interactions and oligomerization of hantavirus glycoproteins. J Virol 84:227–242. doi:10.1128/JVI.00481-09. 19828613 PMC2798430

[10] Battisti AJ, Chu YK, Chipman PR, Kaufmann B, Jonsson CB, Rossmann MG 2011 Structural studies of Hantaan virus. J Virol 85:835–841. doi:10.1128/JVI.01847-10. 21068243 PMC3020021

[11] Huiskonen JT, Hepojoki J, Laurinmaki P, Vaheri A, Lankinen H, Butcher SJ, Grunewald K 2010 Electron cryotomography of Tula hantavirus suggests a unique assembly paradigm for enveloped viruses. J Virol 84:4889–4897. doi:10.1128/JVI.00057-10. 20219926 PMC2863824

[12] Li S, Rissanen I, Zeltina A, Hepojoki J, Raghwani J, Harlos K, Pybus OG, Huiskonen JT, Bowden TA 2016 A molecular-level account of the antigenic hantaviral surface. Cell Rep 15:959–967. doi:10.1016/j.celrep.2016.03.082. 27117403 PMC4858563

[13] Willensky S, Bar-Rogovsky H, Bignon EA, Tischler ND, Modis Y, Dessau M 2016 Crystal structure of glycoprotein C from a hantavirus in the post-fusion conformation. PLoS Pathog 12:e1005948. doi:10.1371/journal.ppat.1005948. 27783673 PMC5081248

[14] Guardado-Calvo P, Bignon EA, Stettner E, Jeffers SA, Perez-Vargas J, Pehau-Arnaudet G, Tortorici MA, Jestin JL, England P, Tischler ND, Rey FA 2016 Mechanistic insight into bunyavirus-induced membrane fusion from structure-function analyses of the hantavirus envelope glycoprotein Gc. PLoS Pathog 12:e1005813. doi:10.1371/journal.ppat.1005813. 27783711 PMC5082683

[15] Koch J, Liang M, Queitsch I, Kraus AA, Bautz EK 2003 Human recombinant neutralizing antibodies against Hantaan virus G2 protein. Virology 308:64–73. doi:10.1016/S0042-6822(02)00094-6. 12706090

[16] Heiskanen T, Lundkvist A, Soliymani R, Koivunen E, Vaheri A, Lankinen H 1999 Phage-displayed peptides mimicking the discontinuous neutralization sites of Puumala hantavirus envelope glycoproteins. Virology 262:321–332. doi:10.1006/viro.1999.9930. 10502511

[17] Horling J, Lundkvist A 1997 Single amino acid substitutions in Puumala virus envelope glycoproteins G1 and G2 eliminate important neutralization epitopes. Virus Res 48:89–100. doi:10.1016/S0168-1702(97)01436-6. 9140197

[18] Cifuentes-Munoz N, Darlix JL, Tischler ND 2010 Development of a lentiviral vector system to study the role of the Andes virus glycoproteins. Virus Res 153:29–35. doi:10.1016/j.virusres.2010.07.001. 20619306

[19] Schmaljohn CS, Chu YK, Schmaljohn AL, Dalrymple JM 1990 Antigenic subunits of Hantaan virus expressed by baculovirus and vaccinia virus recombinants. J Virol 64:3162–3170. 1972201 10.1128/jvi.64.7.3162-3170.1990 PMC249520

[20] Choi Y, Kwon YC, Kim SI, Park JM, Lee KH, Ahn BY 2008 A hantavirus causing hemorrhagic fever with renal syndrome requires gC1qR/p32 for efficient cell binding and infection. Virology 381:178–183. doi:10.1016/j.virol.2008.08.035. 18834607

[21] Gavrilovskaya IN, Shepley M, Shaw R, Ginsberg MH, Mackow ER 1998 β3 integrins mediate the cellular entry of hantaviruses that cause respiratory failure. Proc Natl Acad Sci U S A 95:7074–7079. doi:10.1073/pnas.95.12.7074. 9618541 PMC22743

[22] Krautkramer E, Zeier M 2008 Hantavirus causing hemorrhagic fever with renal syndrome enters from the apical surface and requires decay-accelerating factor (DAF/CD55). J Virol 82:4257–4264. doi:10.1128/JVI.02210-07. 18305044 PMC2293039

[23] Riblett AM, Blomen VA, Jae LT, Altamura LA, Doms RW, Brummelkamp TR, Wojcechowskyj JA 2015 A haploid genetic screen identifies heparan sulfate proteoglycans supporting Rift Valley fever virus infection. J Virol 90:1414–1423. doi:10.1128/JVI.02055-15. 26581979 PMC4719632

[24] Jin M, Park J, Lee S, Park B, Shin J, Song KJ, Ahn TI, Hwang SY, Ahn BY, Ahn K 2002 Hantaan virus enters cells by clathrin-dependent receptor-mediated endocytosis. Virology 294:60–69. doi:10.1006/viro.2001.1303. 11886265

[25] Halldorsson S, Behrens AJ, Harlos K, Huiskonen JT, Elliott RM, Crispin M, Brennan B, Bowden TA 2016 Structure of a phleboviral envelope glycoprotein reveals a consolidated model of membrane fusion. Proc Natl Acad Sci U S A 113:7154–7159. doi:10.1073/pnas.1603827113. 27325770 PMC4932967

[26] de Boer SM, Kortekaas J, Spel L, Rottier PJ, Moormann RJ, Bosch BJ 2012 Acid-activated structural reorganization of the Rift Valley fever virus Gc fusion protein. J Virol 86:13642–13652. doi:10.1128/JVI.01973-12. 23035232 PMC3503025

[27] Song G 1999 Epidemiological progresses of hemorrhagic fever with renal syndrome in China. Chin Med J (Engl) 112:472–477. 11593522

[28] Yan L, Fang LQ, Huang HG, Zhang LQ, Feng D, Zhao WJ, Zhang WY, Li XW, Cao WC 2007 Landscape elements and Hantaan virus-related hemorrhagic fever with renal syndrome, People's Republic of China. Emerg Infect Dis 13:1301–1306. doi:10.3201/eid1309.061481. 18252099 PMC2857277

[29] Sievers F, Wilm A, Dineen D, Gibson TJ, Karplus K, Li W, Lopez R, McWilliam H, Remmert M, Soding J, Thompson JD, Higgins DG 2011 Fast, scalable generation of high-quality protein multiple sequence alignments using Clustal Omega. Mol Syst Biol 7:539. doi:10.1038/msb.2011.75. 21988835 PMC3261699

[30] Krissinel E, Henrick K 2007 Inference of macromolecular assemblies from crystalline state. J Mol Biol 372:774–797. doi:10.1016/j.jmb.2007.05.022. 17681537

[31] Acuna R, Bignon EA, Mancini R, Lozach PY, Tischler ND 2015 Acidification triggers Andes hantavirus membrane fusion and rearrangement of Gc into a stable post-fusion homotrimer. J Gen Virol 96:3192–3197. doi:10.1099/jgv.0.000269. 26310672

[32] Kleinfelter LM, Jangra RK, Jae LT, Herbert AS, Mittler E, Stiles KM, Wirchnianski AS, Kielian M, Brummelkamp TR, Dye JM, Chandran K 2015 Haploid genetic screen reveals a profound and direct dependence on cholesterol for hantavirus membrane fusion. mBio 6:e00801. doi:10.1128/mBio.00801-15. 26126854 PMC4488941

[33] Petersen J, Drake MJ, Bruce EA, Riblett AM, Didigu CA, Wilen CB, Malani N, Male F, Lee FH, Bushman FD, Cherry S, Doms RW, Bates P, Briley K Jr 2014 The major cellular sterol regulatory pathway is required for Andes virus infection. PLoS Pathog 10:e1003911. doi:10.1371/journal.ppat.1003911. 24516383 PMC3916400

[34] Bowden TA, Bitto D, McLees A, Yeromonahos C, Elliott RM, Huiskonen JT 2013 Orthobunyavirus ultrastructure and the curious tripodal glycoprotein spike. PLoS Pathog 9:e1003374. doi:10.1371/journal.ppat.1003374. 23696739 PMC3656102

[35] Harrison SC 2008 Viral membrane fusion. Nat Struct Mol Biol 15:690–698. doi:10.1038/nsmb.1456. 18596815 PMC2517140

[36] Huiskonen JT, Överby AK, Weber F, Grünewald K 2009 Electron cryo-microscopy and single-particle averaging of Rift Valley fever virus: evidence for GN-GC glycoprotein heterodimers. J Virol 83:3762–3769. doi:10.1128/JVI.02483-08. 19193794 PMC2663282

[37] Sherman MB, Freiberg AN, Holbrook MR, Watowich SJ 2009 Single-particle cryo-electron microscopy of Rift Valley fever virus. Virology 387:11–15. doi:10.1016/j.virol.2009.02.038. 19304307 PMC2673237

[38] Freiberg AN, Sherman MB, Morais MC, Holbrook MR, Watowich SJ 2008 Three-dimensional organization of Rift Valley fever virus revealed by cryoelectron tomography. J Virol 82:10341–13048. doi:10.1128/JVI.01191-08. 18715915 PMC2573222

[39] Dessau M, Modis Y 2013 Crystal structure of glycoprotein C from Rift Valley fever virus. Proc Nat Acad Sci U S A 110:1696–1701. doi:10.1073/pnas.1217780110. PMC3562824 23319635

[40] Kielian M 2006 Class II virus membrane fusion proteins. Virology 344:38–47. doi:10.1016/j.virol.2005.09.036. 16364734

[41] Perez-Vargas J, Krey T, Valansi C, Avinoam O, Haouz A, Jamin M, Raveh-Barak H, Podbilewicz B, Rey FA 2014 Structural basis of eukaryotic cell-cell fusion. Cell 157:407–419. doi:10.1016/j.cell.2014.02.020. 24725407

[42] Guardado-Calvo P, Rey FA 2017 The envelope proteins of the Bunyavirales. Adv Virus Res 98:83–118. doi:10.1016/bs.aivir.2017.02.002. 28433053

[43] Le SQ, Gascuel O 2008 An improved general amino acid replacement matrix. Mol Biol Evol 25:1307–1320. doi:10.1093/molbev/msn067. 18367465

[44] Kumar S, Stecher G, Tamura K 2016 MEGA7: Molecular Evolutionary Genetics Analysis version 7.0 for bigger datasets. Mol Biol Evol 33:1870–1874. doi:10.1093/molbev/msw054. 27004904 PMC8210823

[45] Aricescu AR, Lu W, Jones EY 2006 A time- and cost-efficient system for high-level protein production in mammalian cells. Acta Crystallogr D Biol Crystallogr 62:1243–1250. doi:10.1107/S0907444906029799. 17001101

[46] Elbein AD, Tropea JE, Mitchell M, Kaushal GP 1990 Kifunensine, a potent inhibitor of the glycoprotein processing mannosidase I. J Biol Chem 265:15599–15605. 2144287

[47] Walter TS, Diprose JM, Mayo CJ, Siebold C, Pickford MG, Carter L, Sutton GC, Berrow NS, Brown J, Berry IM, Stewart-Jones GB, Grimes JM, Stammers DK, Esnouf RM, Jones EY, Owens RJ, Stuart DI, Harlos K 2005 A procedure for setting up high-throughput nanolitre crystallization experiments. Crystallization workflow for initial screening, automated storage, imaging and optimization. Acta Crystallogr D Biol Crystallogr 61:651–657. 15930615 10.1107/S0907444905007808 PMC7159505

[48] Winter G 2010 xia2: an expert system for macromolecular crystallography data reduction. J Appl Crystallogr 43:186–190. doi:10.1107/S0021889809045701.

[49] Adams PD, Afonine PV, Bunkoczi G, Chen VB, Davis IW, Echols N, Headd JJ, Hung LW, Kapral GJ, Grosse-Kunstleve RW, McCoy AJ, Moriarty NW, Oeffner R, Read RJ, Richardson DC, Richardson JS, Terwilliger TC, Zwart PH 2010 PHENIX: a comprehensive Python-based system for macromolecular structure solution. Acta Crystallogr D Biol Crystallogr 66:213–221. doi:10.1107/S0907444909052925. 20124702 PMC2815670

[50] Emsley P, Cowtan K 2004 Coot: model-building tools for molecular graphics. Acta Crystallogr D Biol Crystallogr 60:2126–2132. doi:10.1107/S0907444904019158. 15572765

[51] Chen VB, Arendall WB, Headd JJ, Keedy DA, Immormino RM, Kapral GJ, Murray LW, Richardson JS, Richardson DC 2010 MolProbity: all-atom structure validation for macromolecular crystallography. Acta Crystallogr D Biol Crystallogr 66:12–21. doi:10.1107/S0907444909042073. 20057044 PMC2803126

[52] Brown PH, Schuck P 2006 Macromolecular size-and-shape distributions by sedimentation velocity analytical ultracentrifugation. Biophys J 90:4651–4661. doi:10.1529/biophysj.106.081372. 16565040 PMC1471869

[53] Mastronarde DN 2005 Automated electron microscope tomography using robust prediction of specimen movements. J Struct Biol 152:36–51. doi:10.1016/j.jsb.2005.07.007. 16182563

[54] Zheng SQ, Palovcak E, Armache JP, Verba KA, Cheng Y, Agard DA 2017 MotionCor2: anisotropic correction of beam-induced motion for improved cryo-electron microscopy. Nat Methods 14:331–332. doi:10.1038/nmeth.4193. 28250466 PMC5494038

[55] Scheres SH 2012 RELION: implementation of a Bayesian approach to cryo-EM structure determination. J Struct Biol 180:519–530. doi:10.1016/j.jsb.2012.09.006. 23000701 PMC3690530

[56] Rohou A, Grigorieff N 2015 CTFFIND4: Fast and accurate defocus estimation from electron micrographs. J Struct Biol 192:216–221. doi:10.1016/j.jsb.2015.08.008. 26278980 PMC6760662

[57] Heymann JB, Belnap DM 2007 Bsoft: image processing and molecular modeling for electron microscopy. J Struct Biol 157:3–18. doi:10.1016/j.jsb.2006.06.006. 17011211

[58] Pintilie GD, Zhang J, Goddard TD, Chiu W, Gossard DC 2010 Quantitative analysis of cryo-EM density map segmentation by watershed and scale-space filtering, and fitting of structures by alignment to regions. J Struct Biol 170:427–438. doi:10.1016/j.jsb.2010.03.007. 20338243 PMC2874196

[59] Pettersen EF, Goddard TD, Huang CC, Couch GS, Greenblatt DM, Meng EC, Ferrin TE 2004 UCSF Chimera—a visualization system for exploratory research and analysis. J Comput Chem 25:1605–1612. doi:10.1002/jcc.20084. 15264254

[60] Ren J, Wen L, Gao X, Jin C, Xue Y, Yao X 2009 DOG 1.0: illustrator of protein domain structures. Cell Res 19:271–273. doi:10.1038/cr.2009.6. 19153597

[61] Crispin M, Bowden TA, Coles CH, Harlos K, Aricescu AR, Harvey DJ, Stuart DI, Jones EY 2009 Carbohydrate and domain architecture of an immature antibody glycoform exhibiting enhanced effector functions. J Mol Biol 387:1061–1066. doi:10.1016/j.jmb.2009.02.033. 19236877

[62] Brookes E, Demeler B, Rosano C, Rocco M 2010 The implementation of SOMO (SOlution MOdeller) in the UltraScan analytical ultracentrifugation data analysis suite: enhanced capabilities allow the reliable hydrodynamic modeling of virtually any kind of biomacromolecule. Eur Biophys J 39:423–435. doi:10.1007/s00249-009-0418-0. 19234696 PMC2872189

[63] Brookes E, Demeler B, Rocco M 2010 Developments in the US-SOMO bead modeling suite: new features in the direct residue-to-bead method, improved grid routines, and influence of accessible surface area screening. Macromol Biosci 10:746–753. doi:10.1002/mabi.200900474. 20480513

[64] Rai N, Nollmann M, Spotorno B, Tassara G, Byron O, Rocco M 2005 SOMO (SOlution MOdeler) differences between X-ray- and NMR-derived bead models suggest a role for side chain flexibility in protein hydrodynamics. Structure 13:723–734. doi:10.1016/j.str.2005.02.012. 15893663

